# Physiological, Psychosocial and Substance Abuse Effects of Pornography Addiction: A Narrative Review

**DOI:** 10.7759/cureus.33703

**Published:** 2023-01-12

**Authors:** Haseeb Mehmood Qadri, Abdul Waheed, Ali Munawar, Hasan Saeed, Saad Abdullah, Tayyba Munawar, Shaheer Luqman, Junaid Saffi, Awais Ahmad, Muhammad Saad Babar

**Affiliations:** 1 Surgery, Lahore General Hospital, Lahore, PAK; 2 Surgery, Shaikh Zayed Hospital, Lahore, PAK; 3 Pathology, Allama Iqbal Medical College, Lahore, PAK; 4 General Surgery, Allama Iqbal Medical College, Lahore, PAK; 5 Surgery, Jinnah Hospital, Lahore, PAK; 6 General Surgery, Lahore General Hospital, Lahore, PAK

**Keywords:** narrative review, harmful effects of porn, cybersex, compulsive sexual behavior or sexual addiction, porn addiction, pornography 2022

## Abstract

Internet pornography provides explicit content in various forms and can progress from habit to addiction. The consumption of online porn has risen due to the general use of current technology. The main reasons people consume it are sexual arousal and sexual enhancement. We planned this review study to identify the reasons for online pornography utilization, the mechanisms involved in its addiction, and its physiological, emotional, behavioral, social, and substance abuse effects. After a detailed literature search using PubMed Central and Google Scholar, four case studies and nine original articles from 2000 to 2022 were included. The main findings of the literature demonstrated that watching porn was most frequently done out of boredom, for sexual gratification, and to pick up new fashion and behavior ideas from these movies. In all facets of the users' lives, negative consequences were seen. Due to the explosion of new technologies, online pornography has risen to an alarming level, which has very injurious effects on societies and individuals. Therefore, it is high time to get rid of this addiction to protect our lives from its harmful effects.

## Introduction and background

Technological advancement in the 21st century has paved the way for new research in the field of addictive disorders, including highly recognized substance addictions and behavioral addictions. These addictions encompass frequent cravings, lack of control, and neglecting duties despite being aware of their mortifying effects on personality and psychological well-being. Pornography consumption, excessive masturbation, and cybersex come under hyper-sexual disorder, a behavioral disorder [1]. Despite the lack of reliable scientific information, being poorly understood and understudied, assessment of this disorder has been a vital task [1,2]. The prevalence rates range from three to six percent, and its affiliation with shame and extreme distress warrants a thorough examination. Technology and especially the internet, has broken ground and tempted people, encouraging their behavior to develop hyper-sexual addictions like online pornography consumption for self-gratification [1].

There has been a significant increase in the consumption of sexually explicit material, even in places where it is illegal, over the years [2,3]. Porn sites getting more visitors than Amazon, Twitter and Netflix combined shows how alarming this situation is. The increase in online pornography consumption can be appreciated because, in 2019, the world's top porn site's traffic jumped to one billion visitors per month, placing it among the world's top internet sites [2]. Today, 46-74% of men and 16-41% of women watch porn [3]. Various factors have been considered in the striking increase in watching online pornography. Since the outbreak of COVID-19, there has been a significant increase in internet surfing, online shopping, and of course, online pornography due to lockdown and social restrictions [2]. Boredom and loneliness due to these restrictions are essential factors in this regard [2,3]. Considering 'porn' and 'depression' are the most searched topics online during the pandemic, emphasize the vital connection between the former and the latter [2]

Not only has pornography been consumed to temporarily lower stress from being lonely and isolated, but also information seeking, sexual arousal, and enhancement prove to be driving forces towards greater porn consumption [3]. People watch pornographic content to elate their moods and evade routine stressors, but unknowingly pornography itself is a significant stressor. Neurobiological explanations suggest that pornography has dramatically reduced overall sexual activity among couples [2]. A sharp increase in the prevalence of erectile dysfunction (ED) and low sexual desire was observed in men under 40. In a study conducted in 1999, ED and low sexual desire rate was 5%, respectively. By 2011, ED rates have increased to 14-28% in European men between 18-40 years [4]. The following research paper has been formulated to explain the increase in pornography statistics and its impact on physiological, social, and emotional aspects of human life, as well as the drug-related effects of pornography.

## Review

Materials and methods

Literature Search Strategy

The search for this narrative review was done using the database engines PubMed Central and Google Scholar. The search was conducted using the Boolean operators ("AND" and "OR") with the following combination of keywords: "porn", "pornography", "pornography addiction", "pornographic addiction", "social effects of porn", "behavioral effects of porn", "emotional effects of porn ", "drug-related effects of porn", "drug abuse effects of porn" and "substance abuse effects of porn".

Eligibility Criteria

All case reports, case-control studies, randomized controlled trials (RCTs), and cohort studies published from 2000 to 2022 were included. Case studies, data articles, gaming articles, published erratum, review articles, and articles containing non-human sources were all excluded.

Data Extraction

Four reviewers read the full articles independently and extracted the data considering the following variables: title, abstract, methods, and main results. The data were then verified for completeness and accuracy and were harvested into a Microsoft Office Word-generated pro forma.

Study Selection

As a result of the keyword terms in the databases, a total of 300 articles were obtained. For this review, researchers used a literature search strategy outlined in Table 1.

**Table 1:** Literature Search Strategy.

Sr. #	Search Strategy	No. of Publications
1.	Publications retrieved from the PubMed database,	245
2.	Publications retrieved from Google Scholar	55
3.	Total number of publications from the electronic search	300
4.	Total number of publications after removal of duplication	190
5.	Total number of publications available via open access/free full-text form	98
6.	Publications remaining after the title or abstract screening	13
7.	Total included publications	13
8.	Total included research or original articles	9
9.	Total number of case reports	4

Results

Most of the studies were conducted between 2017 and 2022. The most common journals used for these studies were "Archives of Sexual Behavior" and "Frontiers in Psychology". Most studies were conducted in the United States of America (USA) and Spain. The most common study type was prospective (Table 2).

**Table 2:** Summary of Original Articles – Article Details

Study by	Parameters				
	Title	Year	Journal	Country of Study	Study Type
Mark et al. [5]	Enjoyment, exploration, and education: understanding the consumption of pornography among young men with non-exclusive sexual orientations.	2017	SAGE publications	United States of America	Retrospective study
Razwan et al. [6]	Does addiction to online pornography affect the behavioral pattern of undergraduate private university students in Bangladesh?	2018	International journal of health sciences	Bangladesh	Cross-sectional study
López et al. [7]	Audio described vs. audiovisual porn: cortisol, heart rate, and engagement in visually impaired vs. sighted participants.	2021	Frontiers in psychology	Spain	Prospective study
Burtaverde et al. [8]	Why do people watch porn? An evolutionary perspective on the reasons for pornography consumption	2021	Evolutionary psychology	Romania	Prospective study
Jacobs et al. [9]	Associations between online pornography consumption and sexual dysfunction in young men: a multivariate analysis based on an international web-based survey	2021	JMIR public health and surveillance	Belgium	Prospective study
Niki et al. [10]	Porn sex versus real sex: sexual behaviors reported by a U.S. probability survey compared to depictions of sex in mainstream internet‑based male–female pornography	2022	Archives of sexual behavior	United States of America	Retrospective study
Josep et al. [11]	Pornography use in adolescents and its clinical implications	2020	Journal of clinical medicine	Spain	Prospective study
Davide et al. [12]	Pornography use profiles and the emergence of sexual behaviors in adolescence	2021	Archives of sexual behavior	Netherland	Prospective study
Christina et al. [13]	Compulsive internet pornography use and mental health: a cross-sectional study in a sample of university Students in the United States	2021	Frontiers in psychology	United States of America	Cross-sectional study

The most common objective in these studies was to identify the reason for pornography consumption and its effects on specific aspects of one's life. The most common method used in these studies was a questionnaire-based survey or interviews. Mostly inclusion criteria were selecting random individuals from either universities or schools. Most studies did not have exclusion criteria (Table 3).

**Table 3:** Summary of Original Articles – Methodology

Study by	Parameters			
	Objective	Method Used	Inclusion Criteria	Exclusion Criteria
Mark et al. [5]	Understand pornography consumption as a form of leisure activity. Explore the educational benefits of this consumption, related to their sexual desires and identities, and learn new sexual techniques.	The lead author conducted semi-structured, in-depth interviews, averaging 65 minutes, in a private interview room. Questions on pornography included: why they watched it; their attitudes towards it; their history of watching pornography; how they accessed it; frequency of consumption; and their perspectives on how it impacted them. Religious upbringing was not part of the interview schedule but was discussed when raised by participants.	A criterion for eligibility in the study was that participants did not identify as ‘exclusively heterosexual’ or ‘exclusively homosexual’, measured on a Kinsey-type nine-point scale of sexuality.	Participants identified as ‘exclusively heterosexual’ or ‘exclusively homosexual were excluded.
Razwan et al. [6]	To investigate the association between the consumption of online pornography and socio-behavioral patterns among students from a private university in Bangladesh.	299 undergraduate students (70.6% male) at the First Capital University of Bangladesh were interviewed using a structured questionnaire.	The study participants were recruited, inclusive, between 03 April and 10 May 2016.	Nil
López et al. [7]	Impact of audio description on porn reception	Participants were allocated into three experimental groups: – UM-AV: 25 students watched and heard the scenes in their audiovisual version. – UM-AD: 22 students listened to the audio-described version without images (original audio + AD, black screen). – once: 22 visually impaired participants listened to the audio-described version (original audio + AD, black screen). Two porn scenes were selected as stimuli for the experiment. Clips were treated as one 12-min video to give enough time for the physiological reactions to unveil. The two scenes were presented in randomized order. Instruments Five self-report questionnaires were used to measure the participants’ subjective feeling component of emotion.	The criterion for inclusion in the one is having, at least, one of the following visual conditions in both eyes and a reliable prognosis of no visual improvement: (a) visual acuity equal to or less than 0.1 obtained with the best possible optical correction, (b) visual field reduced to 10 degrees or less.	Nil
Burtaverde et al. [8]	Reasons for pornography consumption using a bottom-up approach (i.e., open-ended questionnaire)	Study 1: why do people say they watch porn? 276 undergraduate psychology students were asked: “why do you watch pornography?” All the participants were asked to list the three most important reasons for watching pornography; Study 2: personality and patterns of pornography consumption 322 undergraduate students were asked to rate the importance of each of the 78 reasons identified in Study 1. Demographic data were also collected. Socio-sexuality, used as an indicator of short-term mating orientation, was measured; Study 3: Confirmatory factor analysis and convergent validity. Identified reasons were used for watching pornography, consisting of 78 items, for measuring reasons for pornography consumption. Four sexual fantasies and eight dimensions of sexual disgust were also measured.	Endorsement of pornography consumption, assessed via one item (i.e., “do you watch pornography?”). Those who endorsed Burta˘verde (Burtaverde) et al. 3 this item was included in the study.	Nil
Jacobs et al. [9]	How pornography consumption correlates with sexual functioning in young men.	A 118-item questionnaire was developed, including demography, medical history, alcohol, and drug usage, sexual preferences, ED, masturbation, pornography consumption, and partner satisfaction. An online, Web-based survey was created using the Qualtrics platform and tested several times by the team members. The link to the questionnaire was spread. Data was collected to evaluate the association between PPC and ED.	Nil	Nil
Niki et al. [10]	Compare the prevalence of survey respondents’ event-level sexual behaviors with those depicted in mainstream pornography online videos; compare event-level condom use with condom use in pornographic videos; event-level orgasm with a prevalence of orgasms in pornographic videos; and assess whether respondents’ partnered use of pornography was associated with the sexual behaviors in which they report engaging.	This study utilized two large and distinct datasets. The sample of Americans’ most recent sexual experiences is from the 2014 national survey of sexual health and behavior (NSSHB-Wave 4)	Individuals were randomly selected and recruited via mailing and telephone to join the panel.	Nil
Josep et al. [11]	To explore whether dispositional, developmental, and social variables predict pornography use and to evaluate whether these variables not only predict pornography use but also moderate the extent to which pornography use predicts criterion variables.	Survey-based research.	Nil	Nil
Davide et al. [12]	To explore adolescent pornography consumption and its association with sexual development in early and middle adolescence.	Data for this study were collected as longitudinal research via a questionnaire from school-going adolescents.	Nil	Nil
Christina et al. [13]	To explore the potential relationship between compulsive use of pornography and mental health in university students.	An anonymous survey was sent via the university Student‘s email address to all students taking classes at Franciscan University.	Before completing the survey, participants aged 18 years and above were directed to a consent form to analyze and publish the overall results.	Any individual younger than 18 years of age, not a student at the Franciscan University of Steubenville, who responded “No” or did not complete the consent question and who did not complete the survey question regarding their age and did not provide a response for the last time they viewed pornography were excluded from the study.

Most of the studies had a sample size of less than 1000. Most studies preferred observing the adult population's behavior and patterns, and the chi-square test was frequently utilized for statistical analysis. Key results of these studies showed that the common reasons to watch pornography were boredom, seeking sexual pleasure, and learning new trends and behavior used in such films. It was also noticed that watching such content had tremendous effects on all aspects of their lives, mainly detrimental. No specific clinical diagnoses were made in these studies (Table 4).

**Table 4:** Summary of Original Articles – Materials

Study by	Parameters			
	Studied Sample	Studied Population	Statistical Test Used	Key Results
Mark et al. [5]	Male, aged between 18 and 32.	Adolescents and adults	Nil	Participants watched pornography from an average self-reported age of first consumption, being 14 years old and the youngest. All participants used porn as a means of sexual gratification. Some participants watched it with others as a bonding activity, relieving boredom, and an educational tool to understand their sexual identities and explore new sexual activities and techniques.
Razwan et al. [6]	1,500 undergraduate students.	Adults	Chi-square test	The use of pornography was significantly higher among students who gathered late at night with their friends, frequently argue/fight with them, frequently fool around with them, and did not go to bed on time.
López et al. [7]	69 Spanish participants.	Only adult population	ANOVAs	No significant association between pornography consumption and salivary cortisol. Heart rate results did not report a significant main effect of the time factor.
Burtaverde et al. [8]	Study I 276 Study 2 322 Study 3 327	Adult population only	Kaiser-Meyer-Olkin test (KMO), the “screen test,” and Horn’s parallel analysis.	Study 1 In the case of men, high sex drive, learning about sex and improving sexual performance, and regulating mood and emotions were the main reasons identified. For women, we concluded that they could be organized on the same three general themes. Study 2 All the extracted factors explained 57.17% of the total variance. Study 3 Individuals with higher scores on the first factor also have higher scores on the second and third factors. Individuals with higher scores on the second factor also have higher scores on the third and lower scores on the fourth. People with high scores on the third factor.
Jacobs et al. [9]	5770 men	Adult Population only	Nonparametric tests (chi-square, Kruskal Wallis, and Mann-Whitney-U) were used for the univariate analyses.	Problematic Pornography Consumption (PPC). There was a statistically significant (P<.001) difference between the median cypat score for those who attempted penetrative sex in past weeks vs. did not have erectile dysfunction according to their iief-5 scores of our sexually active participants had some degree ed classified as having needed watch more or extreme pornography achieve same level arousal compared with experience this need. mild-moderate: moderate: severe:
Niki et al. [10]	Of 4596 individuals screened, 2098 (45.6%) completed the entire survey	Adults	Pearson’s chi-squared tests	Porn sex is different from real sex. Additionally, this study found a relationship between dyadic viewing of pornography and event-level sexual repertoire in ways that suggest viewing pornography with a partner may be associated with sexual variety and orgasm.
Josep et al. [11]	1500 participants.	Adults	Stata16 for Windows	Knowing adolescent pornography consumers’ profiles and the impact of pornography on this population would allow for designing more effective prevention and regulation proposals.
Davide et al. [12]	668 project STARS participants	Pre-adolescents	Chi-square (for categorical variables) Kruskal–Wallis (for continuous variables)	Our findings corroborate that sexual media matters for many young adolescents and more so for boys compared to girls.

Most of the studies describe mixed effects of pornography on the adult population. These include physiological effects on sexual variety and orgasm; emotional effects, i.e., satisfaction; social effects, i.e., stigma in friends' circle associated with not watching pornography and less commonly, pornography-induced drug effects (Table 5).

**Table 5:** Summary of Original Articles – Factors Studied

Study by	Factors Studied			
	Physiological Effects of Porn on Body	Emotional Effects of Porn	Porn-induced Drug Abuse Effects	Social Effects of Porn
Mark et al. [5]	No	Yes	No	Yes
Razwan et al. [6]	No	Yes	No	Yes
Lopez et al. [7]	Yes	Yes	No	No
Burtaverde et al. [8]	No	Yes	No	Yes
Jacobs et al. [9]	No	Yes	No	No
Niki et al. [10]	Yes	Yes	No	Yes
Josep et al. [11]	Yes	Yes	Yes	Yes
Davide et al. [12]	Yes	Yes	No	Yes
Christina et al. [13]	No	Yes	Yes	Yes

The majority of the studies revealed the common physiological effects of pornography include risky sexual behaviors, the development of sexual trajectories, and the use of it with a partner to do different sexual acts and to achieve orgasm. Moreover, common emotional effects described in most studies are to relieve boredom, escape negative thoughts, obtain pleasure and sexual gratification, and enhance sexual performance along with the sexual drive. Additionally, most studies showed that common social effects comprise bonding with male friends, spending more time on websites, difficulty stopping watching pornography sites, and not having sex in a while. Though pornography-induced drug abuse is less common than other effects, these are more serious and include the use of substances to do sexual acts that are out of the norm or to reflect these situations (Table 6).

**Table 6:** Summary of Original Articles – Description of Studied Factors

Study by	What findings are described in each study for the above-mentioned factors?			
	Physiological Effects of Porn on the Body	Emotional Effects of Porn	Porn-induced Drug Abuse Effects	Social Effects of Porn
Mark et al. [5]	Nil	Participants used pornography as a means of Sexual gratification, Pleasure to relieve boredom. Increasing productivity is also helpful in intellectually processing their sexual desires.	Nil	All participants consumed pornography individually, and some participants watched it with others as a bonding activity.
Razwan et al. [6]	Nil	Did not go to bed on time and reported greater consumption of pornography.	Nil	Students gather late at night with their friends. They frequently argue/fight with their friends and fool around with them.
Lopez et al. [7]	The analysis of cortisol reactivity to porn disclosed no significant differences between the groups.	Both sighted, and visually impaired participants reported being moderately aroused and immersed or transported by the films, regardless of AV or AD porn exposure.	Nil	Nil
Burtaverde et al. [8]	Nil	Dimensions like: Increased sex drive, Enhancing sexual performance, Social and instrumental reasons, and Lack of relational and emotional skills were associated with individual differences in the dark triad traits, socio-sexuality, mate value, and life history strategies. Apart from “enhancing sexual performance”, men endorsed the reasons for pornography consumption more strongly. Finally, psychopathy mediated the relationship between sex and several reasons to consume pornography.	Nil	Individuals with high scores on the dimension “social and instrumental reasons” also had high levels of Machiavellianism, psychopathy, and socio-sexuality. Such individuals had acceptance of social groups, such as friends.
Jacobs et al. [9]	Nil	ED seen in our study is situational. In a clinical setting, while questioning a young patient presenting with ED, it can be interesting to question erectile function while masturbating with and without pornography consumption separately.	Nil	Nil
Niki et al. [10]	This study found a relationship between dyadic viewing of pornography with a partner may be associated with sexual variety and orgasm.	More involvement in such videos causes higher levels of fantasies which sometimes cause disappointment, as real-life sex is different from what is shown in such videos.	Nil	Men usually make such videos for men, thus ignoring the needs of females. Thus more responsibility lies on men.
Josep et al. [11]	Pornography could lead to more permissive sexual values and changes in sexual behavior, such as increased risky sexual behaviors.	The greater tendency of males to rate sexual stimuli as more pleasant and arousing and to show a stronger neural response derived from exposure to these sexual stimuli.	Substance use can cause individuals to have such sexual behaviors and activities which are out of the norm.	There is a link between exposure to pornography and risky sexual behaviors such as Multiple sexual partners, History of pregnancies Early sexual initiation.
Davide et al. [12]	Higher levels of pornography use are related to the accelerated development of sexual trajectories.	Adolescents may practice what they have seen and learned, and that pornography use and sexual behaviors may reinforce each other over time.	Nil	Pornography is popular among a substantial group of adolescents, particularly boys, and its consumption may affect both boys’ and girls’ sexual behaviors.
Christina et al. [13]	Nil	“Emotional coping” consisted of using pornography when feeling down To escape negative thoughts.	“Extrinsic” factors appeared to reflect situations that involved external influences, including Being with a sexual partner, being peer pressured, and being drunk or feeling the effects of drugs / illicit substances.	“Pre-occupation” included preferring to access the websites instead of spending time with others. Being short of sleep due to being up viewing the websites, thinking about the websites even when not online, and looking forward to the next internet session accessing the websites. Rushing work to access the websites. Preferring to access the websites while neglecting daily obligations and feeling restless, frustrated, or irritated when unable to access the websites. “Dependence was described as finding it difficult to stop using pornography websites, continuing to access the websites despite the intention to stop, thinking that less time should be spent on the pornography websites, and unsuccessfully trying to spend less time on the websites. The first factor was identified as “interoceptive,” reflecting items related to circumstances that primarily involve the individuals themselves and stemming from internal feelings. These included being alone, lonely, bored, and aroused. The second factor, identified as “impotent,” reflected the increased likelihood of pornography use associated with the absence of possibilities to engage in sexual intercourse, specifically, not having sex in a while (no sex) and not finding someone to engage in sexual intercourse with (no one to sex).

Most case reports deal with pornographic addiction and its relation with different entities and were conducted during the year 2014-19 in Indian Subcontinent and published in various journals (Table 7).

**Table 7:** Summary of Case Reports – Article Details

Study by	Parameters				
	Title	Year	Journal	Country	Clinical Diagnosis
Adnan et al. [14]	Pornographic Addiction: Is it a Distinct Entity?	2019	Medical Journal of Dr. D.Y. Patil University	India	Internet addiction
Daniel et al. [15]	Voyeuristic Disorder and Internet Pornography Addiction: A Case Report	2018	Malaysian journal of medicine and health sciences	Malaysia	Voyeuristic Disorder
Avinash et al. [16]	Compulsive Pornography Use in Late Life: A Case Report	2019	Journal of psychosexual health 1(3–4) 275–276, 2019	India	Compulsive Pornography (hypersexual disorder)
Darshan et al. [17]	A case report of pornography addiction with dhat syndrome	2014	Indian journal of psychiatry 56(4), Oct‑Dec 2014	India	Pornography addiction with Dhat syndrome with mild depression.

The majority of the case reports describing pornographic addiction were conducted in males aged 20-40 years and described the common signs of addiction as being low self-esteem, depression, shyness, and lack of appetite. The most common symptoms were losing interest in sex and spending an average of 5 hours watching pornography daily (Table 8).

**Table 8:** Summary of Case Reports – Clinical Presentation

Study by	Parameters			
	Gender	Age	Symptoms	Signs
Adnan et al. [14]	Male	34 years	Disinterest in sex Relative preoccupation with pornography for the past 3 years.	Low mode Depressed look (HAM-D) score: 9
Daniel et al. [15]	Male	22 years	The intense sexual urge to view an unsuspecting female’s exposed buttock.	Low self-esteem, Shyness
Avinash et al. [16]	Male	69 years	Watching pornography videos and images for 4 to 6 hours a day and enjoying the same and even watching the same sometimes in the middle of the night between 3 am to 6 am.	Mini-Mental State Examination score was 29/30
Darshan et al. [17]	Male	28 years	Uncontrollable excessive watching of pornography. The patient lost interest in soft‑core pornography early and moved on to watching lesbianism and gang sex porn for the past 6 years, spending an average of 5 or more hours every day procuring and watching pornographic movies.	Mild depression (feelings of sadness, excessive guilt, unworthiness, decreased self‑esteem, and decreased appetite).

Half the studies conclude that porn does have physiological effects on the human body, while the other half negates this notion. All the studies describe the emotional effects of porn on the body, and most of the studies indicate that there is no porn-induced drug abuse in humans. Most studies conclude that porn affects the social life of humans (Table 9).

**Table 9:** Summary of Case Reports – Factors Studied

Study by	Factors Studied in Case Report			
	Physiological Effects of Porn	Emotional Effects of Porn	Porn-induced Drug Abuse Effects	Social Effects of Porn
Adnan et al. [14]	Yes	Yes	Nil	Yes
Daniel et al. [15]	Yes	Yes	Nil	Yes
Avinash et al. [16]	Nil	Yes	Nil	Yes
Darshan et al. [17]	Yes	Yes	Nil	Yes

Most studies described the physiological effects of porn, such as low mood, depression, decreased self-esteem, decreased appetite, etc. The emotional effects of porn includes: getting irritable when disturbed while watching porn, not being able to quit it even for a day, and watching women undress in real life to fulfill their desires. The social effects of porn include social awkwardness, inability to concentrate at work, decreased family interaction, and increased cybercrimes such as cyberstalking and pedophilia (Table 10).

**Table 10:** Summary of Case Reports – Description of Studied Factors

Study by	What findings are described in each study for the above-mentioned factors?			
	Physiological Effects of Porn on the body	Emotional Effects of Porn	Porn-induced Substance Abuse	Social Effects of Porn
Adnan et al. [14]	Low mood Depression Hopelessness Helplessness	If his time was cut short or he was interrupted by someone, he developed distress and became irritable.	Nil	Being scolded at the workplace and putting his marriage at stake for being unable to control his urges and desires.
Daniel et al. [15]	Some studies focus on monoamines (serotonin, dopamine, and norepinephrine) as evidenced by the impairment of normal sexual functioning in serotonin and dopamine dysregulation.	Just watching pornographic Material was not enough to quench his sexual addiction, which led him to fulfill his desire by watching women undressing in real life.	Nil	Sexually related internet crime seems to be increased. For example, Cyberstalking, Pedophilic grooming, and Online sexual harassment.
Avinash et al. [16]	Nil	Watching such content gave sexual pleasure to the patient, and he felt sexual arousal.	Nil	Ashamed of being judged by society.
Darshan et al. [16]	Mild depression (feelings of sadness, excessive guilt, feeling of unworthiness, decreased self‑esteem, and decreased appetite).	Restless Irritable, and Dysphoric After attempting to get rid of it.	Nil	Least interactive with family social awkwardness.

The most effective therapies for pornographic addiction are Cognitive Behavioral Therapy (CBT), psycho-education, counseling, distraction techniques, and pharmacological therapy such as fluoxetine or desvenlafaxine (Table 11).

**Table 11:** Summary of Case Reports – Description of Adopted Therapies

Study by	Therapies
Adnan et al. [14]	Cognitive behavioral therapy (CBT) Harm reduction therapy and maintaining the daily content viewing log Progressive muscle relaxation and distraction techniques Pharmacological therapy (SSRI tablet sertraline in 3‑month time)
Daniel et al. [15]	Nil
Avinash et al. [16]	Counseling psychoeducation with his wife about how an active sexual life could be maintained post-60 years of age. Fluoxetine 20 mg/day. This was increased to 40 mg/day in a week Eclectic behavior management
Darshan et al. [17]	Reassurance Sex education for his undue concerns about seminal loss. Pharmacological therapy (desvenlafaxine 50 mg titrated to 100 mg at night) Cognitive Behavioral Therapy (CBT)

Discussion

Neuroscience of Pornography Addiction and Compulsive Sexual Behavior

Addiction is a primary, chronic disease of brain reward, motivation, memory, and related circuitry. Dysfunction in these circuits leads to characteristic biological, psychological, social, and spiritual manifestations. This is reflected in an individual pathologically pursuing reward or relief by substance use and other behaviors [18]. Online pornography corresponds to the use of the internet to engage in various gratifying sexual affairs [1]. Internet addiction comprises a heterogeneous spectrum of Internet activities with a potential illness value, such as gaming, shopping, gambling, or social networking [18].

Today, it has been considered that mostly the male population between 20-40 years of age is actively involved in pornography consumption, and factors such as knowledge and curiosity lead a person to this addictive behavior. This factor is comparable to the emotional factors because of the stigma of sexual talk in our society. In contrast, other studies reported that sexual arousal and sexual enhancement were the predominant motivations for pornography consumption among the self-reported reasons. Aside from sexual arousal and enhancement, coping and boredom are linked with greater use of pornography as well [3].

Hypersexual behavior is generally referred to as Compulsive Sexual Behavior (CSB). However, more recent definitions of CSB usually refer to multiple sexual behaviors that can be compulsive: the most commonly reported being masturbation, followed by compulsive use of pornography, promiscuity, compulsive cruising, and multiple relationships (22-76%) [4]. Anxiety disorders, mood disorders, substance use disorders, and sexual dysfunction have all been linked to hypersexual behavior. Pornography is merely one of the acknowledged accessories for hypersexual conduct, which is the subject of the majority of research investigations [1].

Porn users had greater electroencephalography (EEG) readings and less desire for masturbation porn, which is reflected in the difference in erection quality. It is well established that viewing pornography increases brain activity among regular users, particularly in the ventral striatum, which is crucial for anticipating rewards. These changes might cause the inability to restrain one's urges to engage in sexual activities. [1].

Physiological Effects of Pornography Addiction

Premature ejaculation, erectile dysfunction, libido, and arousal dysfunction are the most often reported sexual dysfunctions in the general population. Some unique studies associate chronic masturbation, delayed ejaculation, higher anxiety levels, and sexual dysfunction with increased consumption of pornographic content. In a sample of European heterosexual men who had complained of a problem with decreased libido, Carvalheira et al. examined the association between masturbation and the usage of pornography and sexual desire. It was shown that more than half of the study participants who had noticed a substantial drop in libido over the previous six months of the evaluation had used pornographic materials at least once a week. [19] A study in 2015 on high school students discovered a correlation between frequent porn consumption and poor sexual desire [4].

Psychosocial Effects of Pornography Addiction

Most of the studies also described the psychological effects of porn, such as low mood, depression, decreased self-esteem, decreased appetite, etc. There are higher incidence rates of general anxiety, psychological distress, and decreased emotional bonding with family members among Individuals with pornographic addiction [1,3,20]. Most of the studies have shown that porn addiction is one of the types of substance addiction. Our study highlights that common reasons to watch porn were boredom, new trends and behaviors, and seeking sexual pleasure, similar to the results of other articles as participants used pornography to have sexual gratification as a part of masturbation as pornography consumption is an important tool for mood management and stress relief. Common emotional effects described in other studies that are linked with greater consumption of pornography involve emotional coping with social isolation, loneliness, and stress, to escape negative thoughts, to enhance sexual performance along with the sexual drive [3].

Pornography addiction is related to significant psychological and socio-functional impairment [20]. Long-term use of online pornography leads to direct and proportional consequences of engaging in maladaptive behavior. Pornography use has been associated with sexual dissatisfaction, which leads to having more sexual partners and engaging in paid sex. This is noted in relationships which are usually one-sided. [1] Another reason for the increase porn consumption may be that people use sex as a survival mechanism for dealing with depressive symptoms, loneliness, and even fear of death. During this time, coronavirus-themed porn might be viewed as an eroticization of fear [3]. Frequent viewers of sexually explicit material had a more liberal or positive attitude towards it and believed that using it could create a more stimulating sex life [20].

Pornography provides temporary relief from negative thoughts like anxiety and stress. With the easy availability of the internet and the emergence of computer technologies, pornography has become an essential economic venture [3,19]. Still, most studies conclude that porn affects the social life of humans. These include social effects, i.e., stigma in friends circle associated with not watching pornography. Most studies showed that common social effects comprise bonding with male friends, spending more time on websites, and difficulty in controlling oneself from watching pornography sites. The social effects of porn include social awkwardness, not being able to concentrate at work, decreased family interaction, and increased cyber crimes such as cyber stalking and pedophilia.

Substance Abuse Effects of Pornography Addiction

Alcohol consumption and being under the influence of addictive drugs alter the sexual behavior of humans. Pornographic consumption stimulates the brain's reward system as those in drug addicts, reflecting the relationship between drug addiction and pornographic addiction [20]. A study found that 9.80% of the drug addicts studied had pornographic addiction as well [1]. However, past studies have not shown a definite link to whether pornographic addiction increases drug abuse. The changes found in the brain of substance abusers have been compared to the changes in pornographic addiction, including; sensitization, desensitization, hypo-frontality, and Dysfunctional stress systems as evidenced by magnetic resonance imaging (MRI) and electroencephalography (EEG) [1]. More research is needed to establish a link between drug abuse in pornographic addiction and vice versa.

We analyze that there needs to be agreement on how detrimental it is to consume pornographic material. In this age of technology and the internet, it is imperative to address the sexual, psychological, and behavioral issues brought on by minors' easy access to online pornographic content and anonymity. Pornography should not be prohibited when used in moderation for amusement during free time or by couples to increase their sexual satisfaction. However, excessive use that causes avoidance, emotional issues, anxiety, and depression should be addressed. It is essential to identify and address the fundamental causes of pornography addiction, which frequently include boredom, loneliness, strained relationships, despair, etc. It is advisable to see therapists who view this as an issue. Physical activity is incorporated into daily living to reduce consumption and dramatically benefits a person's healthy relationships. The typical time spent watching porn decreases with a healthy routine, early bedtime, and limited late-night viewing.

## Conclusions

Internet addiction includes various online behaviors that may have medical implications. Concurrently, the development of new technology has increased problematic addictive behavior, particularly addiction to online pornography. The most often reported sexual dysfunctions in the general population are premature ejaculation, erectile dysfunction, and desire and arousal dysfunction. The reward system's functioning, as well as the brain connections that control erection and sexual desire, may change as a result of regular exposure to porn, which may cause a delay in ejaculation. Hypersexual behavior has been associated with anxiety problems, mood disorders, substance use disorders, and sexual dysfunction. Low mood, melancholy, lower self-esteem, and decreased appetite are some of the physiological impacts of porn. Injurious psychological and emotional repercussions from excessive pornography viewing include anxiety, melancholy, and other clinical manifestations like sexual dysfunction and psychosexual dissatisfaction. Online pornography use for an extended period has direct and proportional adverse effects on behavior.

## Appendices

Authors' Contributions:

HMQ: Concept & Study Design, Literature Search, Literature Study, Manuscript Writing & Drafting, Result Analysis and Interpretation, Figure Support, Manuscript Proof-reading, Formatting, Editing, and Supervision;

AW: Literature Study, Data Collection and Extraction, Manuscript Writing & Drafting, Result Analysis, and Interpretation;

AM: Literature Study, Data Collection and Extraction, Manuscript Writing & Drafting, Result Analysis, and Interpretation;

HS: Literature Study, Data Collection and Extraction, Manuscript Writing & Drafting;

SA: Literature Study, Data Collection and Extraction, Manuscript Writing & Drafting;

TM: Literature Study, Data Collection and Extraction, Manuscript Writing & Drafting, Formatting;

SL: Literature Study, Data Collection and Extraction, Manuscript Writing & Drafting;

JS: Literature Study, Data Collection and Extraction, Manuscript Writing & Drafting;

AA: Literature Study, Data Collection and Extraction, Manuscript Writing & Drafting;

MSB: Literature Study, Manuscript Writing & Drafting, Proofreading.
